# Refractory epilepsy responsive to nonspecific immunossupression: autoimmune or auto inflammatory disease? A case report and review of literature

**DOI:** 10.1186/1546-0096-13-S1-P151

**Published:** 2015-09-28

**Authors:** CH Moreira, LO Mendonca, J Kalil, MT Barros, W Passarelli, CL Jorge, A Pontillo, LHM Castro

**Affiliations:** 1 University of São Paulo, Clinical Neurology, São Paulo, São Paulo, Brazil; 2 University of São Paulo, Clinical Immunology and Allergy, São Paulo, São Paulo, Brazil; 3 University of São Paulo, Institute of Biomedical Science, São Paulo, São Paulo, Brazil

## Introduction

Over the past few years, some epilepsies have been found to be associated with immunomediated disorders. The presence of antibodies against intracellular and extracellular proteins especially in refractory epilepsy provides additional evidence of immunomediated diseases in the brain. In refractory epilepsy, autoantibodies against NMDAR, GAD and the VGKC-complex can be found in 2 to 16% of patients. The definition of autoinflammatory diseases was first introduced by Kastner et al in 2009 after the discovery of a homozygous mutation in the gene MEV1, responsible for Mediterranean Fever. After that, the recognition of inflammassome and interleukin 1 as the main actors in the innate immune system responsible for inflammation in these diseases has changed the treatment for some rare disorders.

## Objective

To present the case of a patient that had persistent pleocytosis in the cerebrospinal fluid and seizures refractory to current drugs for epilepsy, with clinical improvement with nonspecific imunossupression. To discuss the aetiology of inflammation in the brain with a review of the literature.

## Methods

Case report and review of the literature with the terms: “refractory epilepsy”; “chronic encephalitis”; “autoimmune epilepsy”; “autoinflammation of the brain”; “immunomediated brain diseases”; “autoinflammatory disease”

## Results

Case report: The patient, A.C.S. female, 19 years old, is a Brazilian girl, that grew up in São Paulo, from non-consanguineous and healthy parents. Seizures started at 2 years old characterized as motor arrest during seconds followed by crying. When she was 13, she dropped after a spell, seeked medical attention and started medication for epilepsy. Seizures are controlled and usually happened during sleep. At 16 years old, seizures increased and began to happen while she was awake. At this point, seizures are characterized as motor arrest, right arm elevation and cephalic version to the left. Despite the use of innumerous antiepileptic drugs in elevated dosages, she maintained refractory seizures (weekly). Extensive complementary epilepsy investigation was performed. Unexpectedly, CSF studies revealed persistent pleocitosis and/or elevated protein. PET showed marked hypometabolism in left cerebral hemisphere, specially in frontal and temporal regions. Head MRI showed inespecific white matter hyperintensities in FLAIR sequences. A panel of known neural auto-antibodies (NMDA, GABA, AMPA, VGKC) and anti GAD were negative. Considering the CSF alterations, the hypothesis of an immunomediated epilepsy was made and we decided empirically treat with intravenous high-dose corticosteroids (metylprednisolone 5 g monthly for 6 months). After the second month, she improved dramatically and had no more seizures. After 6 months, we decided to keep imunosuppresion with azatioprine with excellent response (2 focal seizures in 18 months). CSF results were normal after treatment. Her quality of life and cognition also improved.

## Review of literature

Immunomediated epilepsy is a controversial issue that is gaining more relevance since the discovery of many neural autoantibodies related to paraneoplasic/immunomediated encephalitis. Frequently, immunomediated encephalitis presents with seizures, including status epilepticus, sometimes refractory. In these disorders, seizures are only controlled after treatment with immunotherapy (high dose corticosteroids, gammaglobulin, etc). A considerable portion of chronic refractory epilepsies are of unknown causes. The role of neural autoantibodies in these cases are under investigation. Besides that, to what extent the damage is caused by the antibodies itself or by the inflamatory reaction in the brain is also debatable. In this case, despite negative antibodies, the patient had CSF alterations that could point to a inflammatory process in the CNS. Based on this hypothesis, we treated with high doses corticosteroids and the patient had an excellent response. Until this moment, the only well recognized monogenic autoinflammatory disease with central nervous involvement are cryopirin-asociated periodic syndromes (FCAS, Mucke-Wells and NOMID), mevalonate kinase deficiency (Hyper IgD and mevalonate aciduria) and mutations in the proteasome (CANDLE disease). Salsano et al, 2013, reported an adult patient with chronic meningitis and progressive hearing loss without evidence of mutations in the IL-1 pathway. This patient also demonstrated only IL-6 hypersecretion and clinical improvement with tocilizumab (interleukin 6 blocker). Dutra et al, 2013 also described an adult patient with CNS involvment and clinical manifestation of CAPS that were responsive to interleukin 1 blocker (anankira) with extensive genetic analysis negative.

## Conclusion

We propose that this patient, with refractory epilepsy and CSF chronic inflamation might be a prototype of a novel category of autoinflammatory disease.

## Consent to publish

Written informated consent for publication of their clinical details was obtained from the patient/parent/guardian/relative of the patient.

